# The association between various smoking behaviors, cotinine biomarkers and skin autofluorescence, a marker for advanced glycation end product accumulation

**DOI:** 10.1371/journal.pone.0179330

**Published:** 2017-06-20

**Authors:** Robert P. van Waateringe, Marjonneke J. Mook-Kanamori, Sandra N. Slagter, Melanie M. van der Klauw, Jana V. van Vliet-Ostaptchouk, Reindert Graaff, Helen L. Lutgers, Karsten Suhre, Mohammed M. El-Din Selim, Dennis O. Mook-Kanamori, Bruce H. R. Wolffenbuttel

**Affiliations:** 1 Department of Endocrinology, University of Groningen, University Medical Center Groningen, Groningen, The Netherlands; 2 Department of Biostatistics, Epidemiology and Scientific Computing, Epidemiology Section, King Faisal Specialist Hospital and Research Centre, Riyadh, Saudi Arabia; 3 Department of Physiology and Biophysics, Weill Cornell Medical College, Doha, Qatar; 4 Department of Internal Medicine, Medical Center Leeuwarden, Leeuwarden, The Netherlands; 5 Bioinformatics Core, Weill Cornell Medical College, Doha, Qatar; 6 Research Centre for Environmental Health, Helmholtz Zentrum Munchen, Neuherberg, Germany; 7 Department of Dermatology, Hamad Medical Corporation, Doha, Qatar; 8 Department of Clinical Epidemiology, Leiden University Medical Center, Leiden, the Netherlands; University of Colorado Denver School of Medicine, UNITED STATES

## Abstract

**Background:**

Skin autofluorescence, a biomarker for advanced glycation end products (AGEs) accumulation, has been shown to predict diabetes-related cardiovascular complications and is associated with several environmental and lifestyle factors. In the present study, we examined the association between various smoking behaviors and skin autofluorescence, as well as the association between several cotinine biomarkers and skin autofluorescence, using both epidemiological and metabolomics data.

**Methods:**

In a cross-sectional study, we evaluated participants from the LifeLines Cohort Study and the Qatar Metabolomics Study on Diabetes (QMDiab). In the LifeLines Cohort Study smoking behavior and secondhand smoking were assessed in 8,905 individuals including 309 individuals (3.5%) with type 2 diabetes. In QMDiab, cotinine biomarkers were measured in saliva, plasma and urine in 364 individuals of whom 188 (51%) had type 2 diabetes. Skin autofluorescence was measured non-invasively in all participants using the AGE Reader.

**Results:**

Skin autofluorescence levels increased with a higher number of hours being exposed to secondhand smoking. Skin autofluorescence levels of former smokers approached levels of never smokers after around 15 years of smoking cessation. Urinary cotinine N-oxide, a biomarker of nicotine exposure, was found to be positively associated with skin autofluorescence in the QMDiab study (p = 0.03).

**Conclusions:**

In the present study, we have demonstrated that secondhand smoking is associated with higher skin autofluorescence levels whereas smoking cessation has a beneficial effect on skin autofluorescence. Finally, urinary cotinine N-oxide might be used as an alternative way for questionnaires to examine the effect of (environmental) tobacco smoking on skin autofluorescence.

## Introduction

Advanced glycation end products (AGEs) are the final products of non-enzymatic glycation and oxidative reactions [1] and comprise a group of irreversibly modified proteins, lipids and nucleic acids [2]. AGEs form stable structures with—and accumulate in—tissues as a result of aging [3,4]. The formation and accumulation of AGEs is increased in conditions such as diabetes and renal insufficiency [5,6]. AGE accumulation can be measured non-invasively in the skin with a device known as the AGE Reader (Diagnoptics Technologies, Groningen, The Netherlands) [7]. Previous studies have demonstrated that higher skin autofluorescence (SAF) is associated with, and a good predictor of the development of cardiovascular morbidity and mortality in patients with diabetes and (end-stage) renal failure [8–10]. In addition, recent studies have shown that SAF levels are increased in patients with chronic obstructive pulmonary disease and peripheral artery disease, independent of diabetes status [11,12].

Several studies have shown elevated SAF levels in smokers compared to non-smokers [9,13]. Moreover, we have recently found both current smoking and the number of pack-years to be strongly associated with higher SAF levels in a large-scale general population [14]. Active and passive tobacco smoking increase the risk of cardiovascular disease and type 2 diabetes, while higher SAF levels are associated with both conditions. As tobacco smoke has been reported to be an exogenous source of AGEs, its accumulation might be considered as an underlying mechanism leading to cardiovascular disease and type 2 diabetes [15].

In studies examining the risk of tobacco smoking on disease outcome, smoking behavior is assessed by general questionnaires. Questionnaires regarding tobacco use are prone to underestimation and reporting biases [16]. Furthermore, the majority of these questionnaires do not include questions regarding secondhand smoking. An alternative way to examine exposure to tobacco smoke is through assessment of biomarkers for tobacco smoke, measured in either urine, saliva or blood [17].

Cotinine, the main metabolite of nicotine, is considered to be a valid biomarker for environmental tobacco smoke exposure due to its high sensitivity, specificity and long half-life time [18,19]. Since secondhand smoking might be underestimated using questionnaires, it would be interesting whether SAF is a more accurate and reliable measure to detect—long-term—exposure to secondhand smoking.

The aim of the present study was to examine the effect of both active and secondhand smoking as well as smoking cessation on SAF. In addition, using metabolomics data we also assessed the association between several cotinine biomarkers and SAF.

## Materials and methods

### Study design

For this cross-sectional study, we used data from two different populations, the Dutch LifeLines Cohort Study and Qatar Metabolomics Study on Diabetes (QMDiab). The LifeLines Cohort Study is a multidisciplinary prospective population-based cohort study with a unique three-generation design that examines the health and health-related behaviors of 8,905 participants in the northern part of The Netherlands [20]. For the current study, we included all participants from Western European origin between 18 and 80 years old with both SAF measurements and information regarding smoking behavior, who participated in our previous studies regarding (genetic) determinants of SAF [14,21]. Exclusion criteria were missing data for smoking status (n = 104) and severely impaired renal function (serum creatinine >140 micromol/L (n = 14) leaving 8,905 individuals, of whom 309 (3.5%) with type 2 diabetes, available for analysis. Before participating in the study, all participants provided written informed consent. The study protocol was approved by the medical ethical review committee of the University Medical Center Groningen, The Netherlands.

The Qatar Metabolomics Study on Diabetes (QMDiab) [22] is a large collaborative effort between the Dermatology Department of Hamad Medical Center (HMC) in Doha, Qatar and Weill Cornell Medical College—Qatar (WCMC-Q).

Subjects were enrolled between February 2012 and June 2012. Before participating in the study, all participants provided written informed consent. Ethical approval was obtained from the Institutional Review Board from both HMC and WCMC-Q. In total, 374 subjects above the age of 18 participated in the study. Smoking data were missing from ten participants, leaving 364 participants of whom 185 (51%) with type 2 diabetes for analyses.

### Data collection

Information regarding ethnicity and smoking behavior was obtained from questionnaires. In QMDiab, ethnicity was determined based on the birthplace of the participant, both parents and four grandparents as described previously [23]. Data on smoking behavior was collected by a detailed self-administered questionnaire. Subjects were classified according to their smoking status as never smoker, former smoker or current smoker (types of tobacco: cigarette, cigarillo, cigar, pipe tobacco). Never smokers were those who had not smoked during the last month and had never smoked for longer than a year. Former smokers were defined as those who had reported smoking for more than a whole year and who had not smoked during the last month and had stopped smoking. Those who had smoked for longer than a year and had not stopped smoking were classified as current smoker. Estimation of total tobacco use of current smokers and their classification into light, moderate and heavy smokers were estimated based on the following quantities: one cigarette = 1 gram tobacco. Light smoking was defined as 10 gram/day or less, moderate as 11 to 20 gram/day and heavy as more than 20 gram/day. Pack-years of smoking was calculated as the number of packs of cigarettes smoked per day multiplied by the number of years a subject has smoked. Finally, information about the age at start and quitting smoking as well as whether a subject have been exposed to secondhand smoking at home was obtained by the questionnaire.

Diagnosis of diabetes mellitus was established at their LifeLines baseline visit either by a single fasting blood plasma glucose level ≥7.0 mmol/L, or when participants reported to have diabetes which was checked with their medication use (i.e. use of oral blood-glucose lowering agents and/or insulin).

### Anthropometry

Using standardized protocols, trained technicians measured body weight with the participant wearing light clothing and without shoes with a 0.1 kg precision. Height was measured without shoes to the nearest 0.5 cm. Body Mass Index (BMI) was calculated as weight (kg) divided by height squared (m^2^).

### Biochemical measures

Blood was collected in the fasting state between 8.00 and 10.00 a.m. and transported to the LifeLines laboratory facility at room temperature or at 4°C, depending on the sample requirements. On the day of collection, fasting blood glucose was measured using a hexokinase method. HbA1c (EDTA-anticoagulated) was analyzed using a turbidimetric inhibition immunoassay on a Cobas Integra 800 CTS analyzer (Roche Diagnostics Nederland BV, Almere, The Netherlands). Creatinine clearance was calculated using the Cockcroft-Gault formula [24].

Cotinine was used as a biomarker for environmental tobacco smoke exposure. Cotinine was measured in non-fasting saliva, plasma and urine specimens which were collected and processed using standardized protocols [22]. In brief, saliva was obtained using the Salivette system following the manufacturer’s recommendations (Sarstedt, Germany). After collection, samples were stored on ice for transportation.

Within six hours after sample collection, all samples were centrifuged at 2,500g for 10 minutes, aliquoted, and stored at -80°C.

Metabolic profiling was achieved using ultra-high-performance liquid-phase chromatography (UHPLC) and gas-chromatography separation, coupled with tandem mass spectrometry (GCMS) at Metabolon Inc. using established procedures (Durham, NC, USA) [25]. The units represent ion counts as measured by the mass spectrometer, which represent semi-quantitative values. In total, 1,568 different metabolites were detected. Osmolality in saliva and urine was measured for normalization purposes.

### AGE reader

For both study groups, SAF was measured with the AGE Reader (Diagnoptics Technologies, the Netherlands). This method has been described in detail previously [7,13]. SAF measures the accumulation of AGEs in the skin which relationship has been demonstrated by measuring AGEs from skin biopsies [7]. The AGE Reader illuminates a skin surface of approximately 4 cm^2^, guarded against surrounding light, with an excitation light source whose wavelength is between 300 and 420nm (peak intensity at ~ 370nm).

Emission light and reflected excitation light from the skin are measured with an internal spectrometer in the range 300 to 600nm. Measurements were performed on the volar side of the forearm, 10cm below the elbow, at room temperature. SAF was calculated by dividing the average emitted light intensity per nanometer in the range of 420–600 nm by the average excitated light intensity per nanometer in the range 300–420 nm and multiplying by 100, and is expressed in arbitrary units (AU). Previous studies have shown an error percentage of around 5–6% when repeated SAF measurement were taken over a single day in control subjects and diabetic patients [7]. The AGE Reader measures SAF also in individuals with a pigmented skin, but only when the UV reflection is above 6%. With a UV reflection below 6% SAF is not given when using the current AGE Reader software.

### Statistical analysis

Data are shown as mean ± standard deviation (SD) or median and interquartile range (IQR) in case of non-normally distributed data. SAF Z-scores were calculated based on the total population. Univariate Analysis of Variance (ANOVA) with a post-hoc Bonferroni test was used to determine differences between the smoking groups. The effect of secondhand smoking (hours per day) on SAF Z scores (adjusted for age, creatinine clearance and diabetes status) was assessed in never smokers and former smokers. We investigated the effect of smoking cessation (in years after smoking abstinence) on SAF (Z-scores) and adjusted in the analysis for age, BMI, creatinine clearance and diabetes status. Multivariable linear regression analysis was performed to examine the association between cotinine biomarkers measured in different specimens and SAF. SPSS (version 22, IBM, Armonk, NY, USA) was used for statistical analysis. A P-value <0.05 was considered statistically significant.

## Results

The clinical characteristics of the LifeLines participants according to their smoking status are shown in Table 1. Mean (±SD) age was 48 ± 12 years in never smokers, 53 ± 11 years in former smokers and 46 ± 10 years for current smokers (p<0.0001). BMI was significantly higher among former smokers compared to never and current smokers (p<0.0001). Current smokers had significantly more pack-years smoked compared to former smokers (16.5 vs 7.7 pack-years, p<0.0001). In former smokers, median time since smoking cessation was 16.7 years (interquartile range 8.0–26.4). Mean SAF levels were significantly higher among current smokers compared to former and never smokers (in non-diabetic subjects, p<0.0001 and in type 2 diabetic individuals, p<0.001). Moreover, in all smoking groups, subjects with type 2 diabetes had significantly higher SAF levels compared to individuals without diabetes (within never smokers, p = 0.001 and within former- and current smokers, p<0.0001).

**Table 1:** Subjects characteristics of the study populations.

**Characteristics LifeLines cohort**	**Never smokers**	**Former smokers**	**Current smokers**
N	3614	3321	1970
Type 2 diabetes/non-diabetes	106 (3) / 3508 (97)	147 (4) / 3174 (96)	56 (3) / 1914 (97)
Age (years)	48 ± 12	53 ± 11***	46 ± 10
Gender (male/female) n (%)	1361 (38) / 2253 (62)	1423 (43) / 1898 (57)	913 (46) / 1057 (54)
Body mass index (kg/m^2^)	26.4 ± 4.4	27.0 ± 4.2 ***	26.1 ± 4.2
Creatinine clearance (ml/min)	113 ± 31	110 ± 31	119 ± 32 ***
Fasting blood glucose			
type 2 diabetes	7.7 ± 1.9	8.2 ± 2.6	7.8 ± 2.4
non-diabetes	5.0 ± 0.5	5.1 ± 0.5 ***	5.0 ± 0.5
HbA1c (%)			
type 2 diabetes	6.7 ± 1.1	7.0 ± 1.2	6.7 ± 1.2
non-diabetes	5.5 ± 0.3	5.6 ± 0.3 ***	5.6 ± 0.3
HbA1c (mmol/mol)			
type 2 diabetes	50.0 ± 12.3	52.7 ± 12.7	50.2 ± 12.9
non-diabetes	36.9 ± 3.3	37.3 ± 3.5 ***	37.3 ± 3.4
Estimated diabetes duration (years)	6.5 (2.9–11.2)	6.6 (3.1–12.3)	4.3 (3.7–7.3)
Duration since stop smoking (years)	n.a.	16.7 (8.0–26.4)	n.a.
Pack-years	n.a.	7.7 (3.2–15.0)	16.5 (9.6–25.0) ***
SAF (AU)			
type 2 diabetes	2.27 ± 0.49	2.48 ± 0.53	2.65 ± 0.61 **
non-diabetes	1.94 ± 0.40	2.09 ± 0.43	2.14 ± 0.48 ***
Age-adjusted SAF Z-scores			
type 2 diabetes	0.05 ± 0.16	0.65 ± 0.16	1.98 ± 0.26 ***
non-diabetes	-0.34 ± 0.02	-0.15 ± 0.03	0.57 ± 0.04 ***
**Characteristics QMDiab cohort**	**Never Smokers**	**Former Smokers**	**Current smokers**
N	269	61	34
Type 2 diabetes/non-diabetes	133 (49) / 136 (51)	35 (57) / 26 (43)	17 (50) / 17 (50)
Age (years)	46 ± 13	51 ± 12	47 ± 11*
Gender (male/female) n (%)	101 (38) / 168 (62)	56 (92) / 5 (8)	29 (85) / 5 (15) *
Body mass index (kg/m^2^)	29.9 ± 6.3	28.4 ± 4.2	28.1 ± 4.8
Ethnicity			
Arab (%)	157 (58)	27 (44)	18 (53)
South Asian (%)	75 (28)	25 (41)	12 (35)
Filipino (%)	27 (10)	6 (10)	3 (9)
Other or mix (%)	10 (4)	3 (5)	1 (3)
Serum creatinine (umol/L)	70 ± 17	89 ± 23 *	77 ± 16
HbA1c (%)			
type 2 diabetes	8.0 ± 1.8	8.3 ± 1.6	8.2 ± 2.4
non-diabetes	5.5 ± 0.4	5.7 ± 0.4	5.7 ± 0.4
HbA1c (mmol/mol)			
type 2 diabetes	64.3 ± 19.4	67.2 ± 17.5	66.5 ± 26.2
non-diabetes	36.6 ± 4.6	38.5 ± 0.3	38.0 ± 4.7
Estimated diabetes duration (years)	10.5 ± 10.0	11.4 ± 10.6	8.7 ± 10.0
Pack-years	n.a.	10.8 ± 14.3	14.0 ± 13.9
Cotinine (saliva, ion counts)	51.4 x 10^4^ ± 45.2 x 10^4^ (n = 23)	34.6 x 10^4^ ± 21.2 x 10^4^ (n = 12)	71.4 x 10^4^ ± 83.8 x 10^4^ (n = 27)
Cotinine (plasma, ion counts)	28.1 x 10^4^ ± 14.4 x 10^4^ (n = 17)	22.3 x 10^4^ ± 13.4 x 10^4^ (n = 14)	35.0 x 10^4^ ± 19.8 x 10^4^ (n = 30)
Cotinine (urine, ion counts)	30.6 x 10^4^ ± 67.8 x 10^4^ (n = 37)	43.6 x 10^4^ ± 42.2 x 10^4^ (n = 20)	81.3 x 10^4^ ± 87.1 x 10^4^ (n = 32) *
Cotinine N oxide (urine, ion counts)	3.0 x 10^4^ ± 4.8 x 10^4^ (n = 88)	4.1 x 10^4^ ± 3.8 x 10^4^ (n = 28)	12.9 x 10^4^ ± 10.1 x 10^4^ (n = 33) ***
Hydroxy-cotinine (urine, ion counts)	191.8 x 10^4^ ± 148.0 x 10^4^ (n = 10)	158.6 x 10^4^ ± 68.1 x 10^4^ (n = 5)	277.8 x 10^4^ ± 275.9 x 10^4^ (n = 14)
SAF (AU)			
type 2 diabetes	2.43 ± 0.72	2.29 ± 0.58	2.49 ± 0.62
non-diabetes	2.10 ± 0.51	2.04 ± 0.58	2.23 ± 0.74
Age-adjusted SAF Z-scores			
type 2 diabetes	0.24 ± 0.25	0.66 ±0.38	0.50 ± 0.67
non-diabetes	-0.10 ± 0.18	-0.72 ± 0.41	-0.04 ± 0.59

Data are presented as means ± standard deviation, or median (interquartile range) and number (%).SAF, Skin autofluorescence; AU, Arbitrary Units.

* p<0.05

** p <0.001

*** p <0.0001

**Fig 1:**
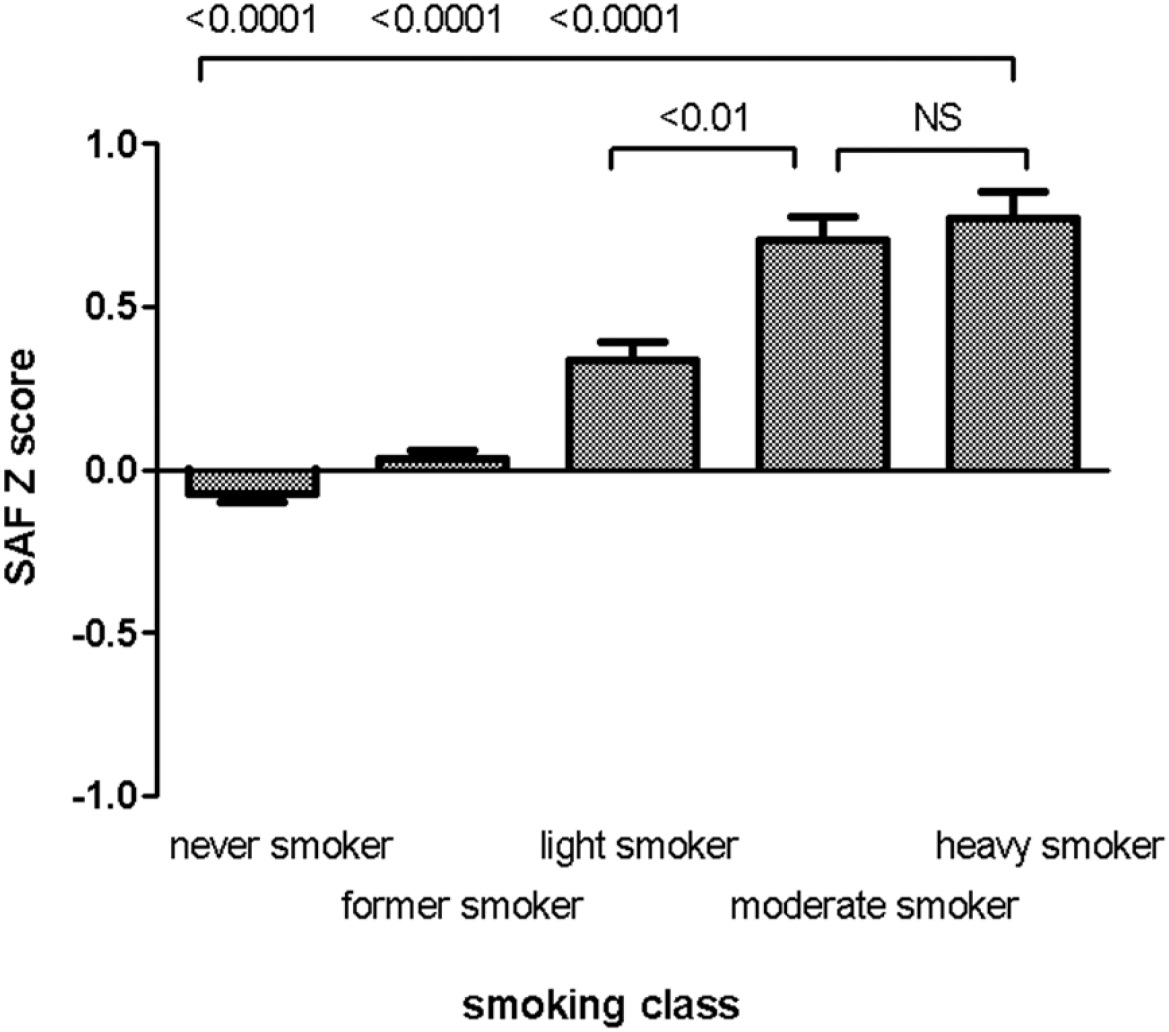
Skin autofluorescence stratified for smoking class (LifeLines Cohort Study). Bars represent mean SAF Z scores (adjusted for age, creatinine clearance and diabetes), whiskers reflect standard error of the mean. Never smoker (n = 3670), Former smoker (n = 3321), Light smoker (0–10 gram tobacco per day, n = 878), Moderate smoker (10–20 gram tobacco per day, n = 537), heavy smoker (>20 gram tobacco per day, n = 475). SAF, skin autofluorescence; AU, arbitrary units; NS, not significant.

Fig 1 shows SAF levels for different smoking classes according to the amount of tobacco smoked per day. SAF levels were highest among heavy smokers compared to light smokers, former and never smokers (p<0.0001). Significantly higher SAF levels were found in moderate vs. light smokers (p<0.01) but not in heavy vs. moderate smokers (p = 0.08).

**Fig 2:**
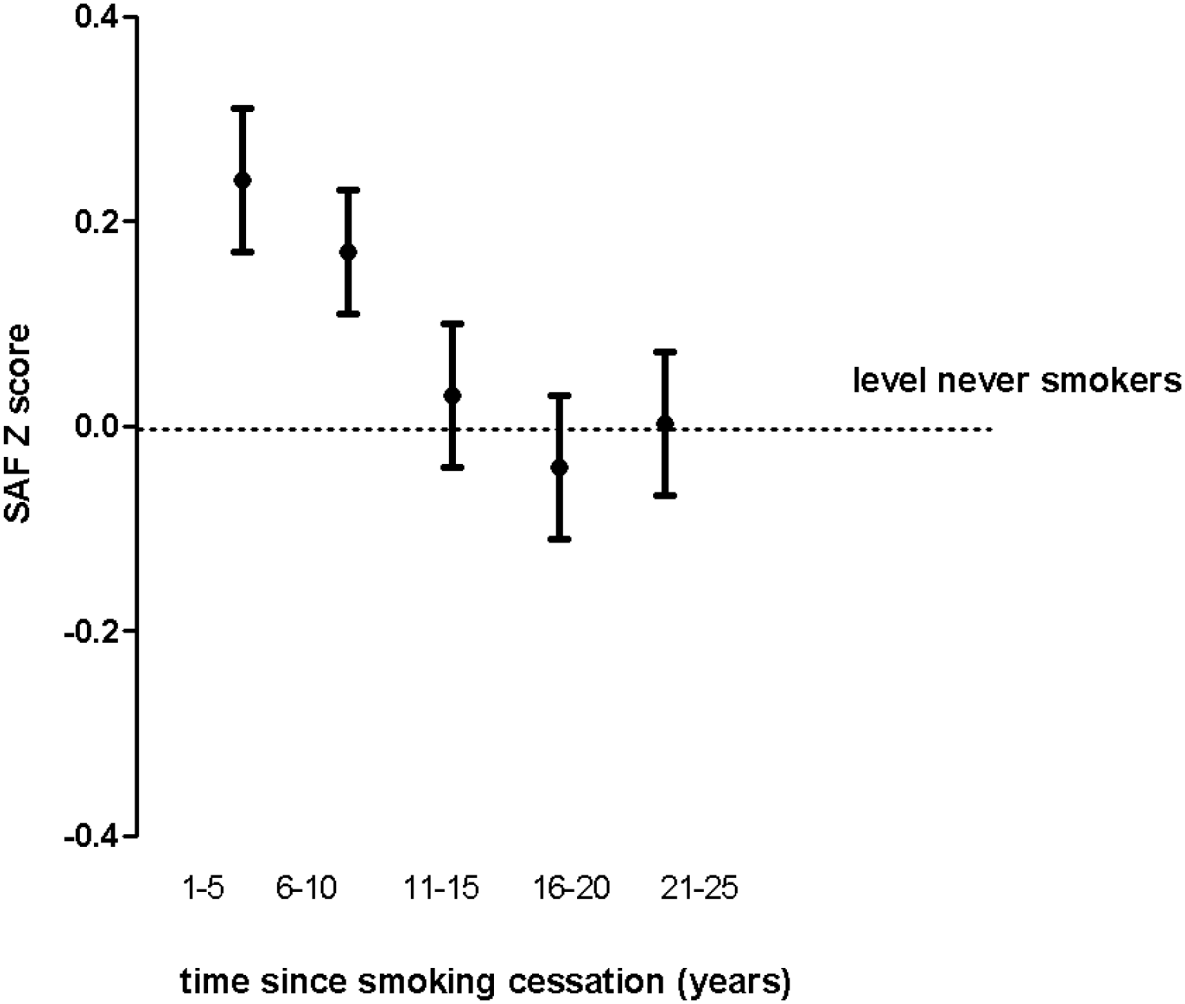
Effect of smoking cessation on skin autofluorescence in former smokers participating in the LifeLines study. Dots show mean SAF Z-scores (adjusted for age, BMI, creatinine clearance and diabetes status). Whiskers reflect standard error of the mean. SAF, skin autofluorescence

The effect of smoking cessation on SAF is shown in Fig 2. SAF Z-scores of former smokers approached levels of never smokers after 15 years of smoking cessation, also after adjusting for age and BMI.

**Fig 3:**
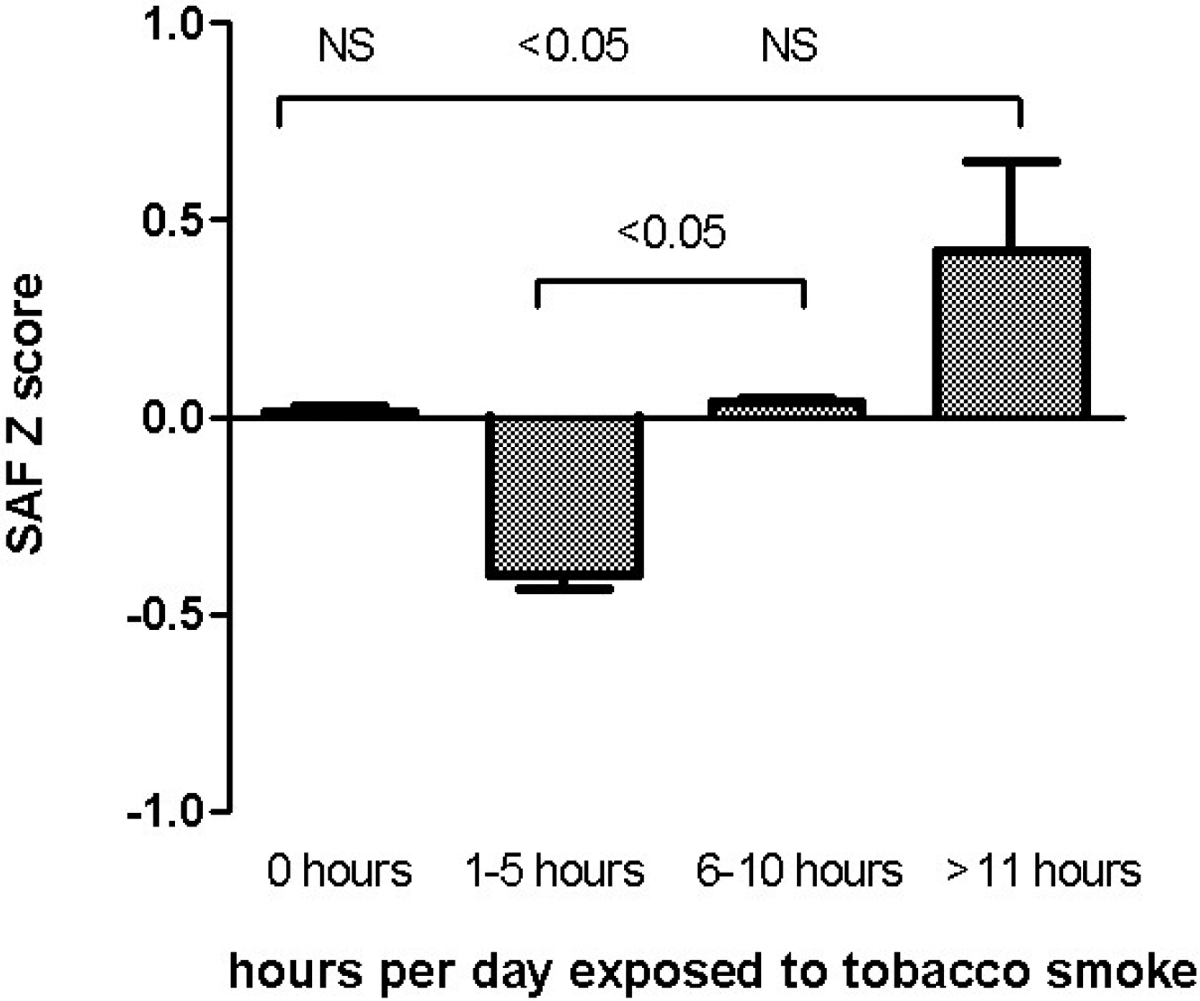
Effect of secondhand smoking on skin autofluorescence in never- and former smokers participating in the LifeLines study. Bars represent mean SAF Z scores (adjusted for age, creatinine clearance and diabetes status) in never smokers and former smokers who stopped smoking for more than 15 years, whiskers reflect standard error of the mean. 0 hours (n = 4213), 1–5 hours (n = 676), 6–10 hours (n = 78), >11 hours (n = 15). SAF, skin autofluorescence; Arbitrary Units, AU; NS, not significant.

At last, we examined the effect of secondhand smoking on SAF (adjusted for age and diabetes status) in never smokers (as well as former smokers who have stopped smoking for more than 15 years, which is based on the previous analysis). A gradual increase in SAF with the number of hours reportedly being exposed to secondhand was observed (Fig 3).

Individuals who had been exposed to secondhand smoking for more than 11 hours per day had significantly higher SAF levels compared to subjects who had been exposed 1–5 hours per day, but not compared to individuals exposed for 6–10 hours or 0 hours per day.

The characteristics of the QMDiab Study participants are shown in Table 1. Mean (±SD) age was 46 ± 13 years in never smokers, 51 ± 12 years among former smokers and 47 ± 11 years in current smokers (p = 0.02). The prevalence of type 2 diabetes was highest among former smokers (57%). There were no significant differences regarding both salivary cotinine and plasma cotinine among the smoking groups. Both urinary cotinine (p<0.05) as well as cotinine N-oxide (p<0.0001) levels were highest for current smokers compared to former and never-smokers.

Five markers for tobacco smoke were evaluated: cotinine in saliva, plasma and urine, and cotinine N-oxide and hydroxy-cotinine in urine. The sensitivity, specificity and the predictive value of the five markers for current smoking are shown in S1 Table. Measured in 148 subjects, cotinine N-oxide had the highest sensitivity (100%) but a relatively low specificity (63.8%) for current smoking.

Cotinine N-oxide was detected in 115 out of 316 individuals (36.4%) who reported to be former or never smoker. However, cotinine in plasma (detected in 61 subjects) had the highest predictive value for current smoking (AUC = 0.94, 95% CI: 0.89, 0.98).

Table 2 shows the results from the multivariable linear regression analyses between smoking variables (smoking status and cotinine biomarkers) and SAF. Current smoking status was significantly associated with higher SAF in the total population. After adjusting for confounders, urinary cotinine N-oxide was found positively and significantly (p = 0.03) associated with SAF in the total population and in individuals without diabetes.

**Table 2:** Smoking status and cotinine markers related to skin autofluorescence using multivariable analyses stratified by diabetes status (QMDiab Study).

	Coefficient Beta (CI)	P value	Coefficient Beta(CI)	P value	Coefficient Beta (CI)	P value
	All subjects		Non-diabetes		Type 2 diabetes	
**Questionnaire**						
Current smoker	0.24 (0.04, 0.45)	**0.02**	0.22 (-0.03, 0.47)	0.08	0.35 (0.00, 0.69)	0.05
Former smoker	0.05 (-0.12, 0.21)	0.59	0.17 (-0.19, 0.22)	0.87	0.08 (-0.19, 0.35)	0.56
**Cotinine markers**						
Cotinine saliva	0.01 (-0.05, 0.07)	0.85	0.02 (-0.04, 0.08)	0.53	-0.02 (-0.15, 0.11)	0.75
Cotinine plasma	0.05 (-0.01, 0.10)	0.12	0.07 (-0.01, 0.15)	0.07	0.04 (-0.05, 0.13)	0.40
Cotinine urine	0.05 (-0.00, 0.11)	0.07	0.06 (-0.01, 0.12)	0.06	0.08 (-0.05, 0.22)	0.23
Cotinine N Oxide (urine)	0.06 (0.01, 0.12)	**0.03**	0.08 (-0.01, 0.15)	**0.03**	0.06 (-0.04, 0.16)	0.21
Hydroxy Cotinine (urine)	0.04 (-0.02, 0.10)	0.17	0.04 (-0.03, 0.10)	0.29	0.05 (-0.05, 0.16)	0.32

Values represent regression coefficients (95% confidence interval) and their corresponding p-values. Model was adjusted for age, gender, ethnicity, body mass index, reflectance, creatinine clearance and the presence of type 2 diabetes. For categorical or dichotomous variables, the effect estimates represent the difference in skin AF compared to the reference group. Markers in saliva and urine were normalized by osmolality and subsequently Z-score normalized to make effect sizes comparable. Thus, effect estimates represent change in SAF per standard deviation increase of cotinine marker. Cotinine metabolites were measured in n = 330 (saliva), n = 358 (plasma), and n = 360 (urine).

## Discussion

In the present study, we combined data from a large population-based cohort study in the Netherlands with metabolomics data from a smaller cohort study in Qatar to examine the effect of smoking intensity, secondhand smoking and smoking cessation on SAF, as well as the association between cotinine biomarkers and SAF.

In our previous study, we have already reported have higher SAF levels in smokers compared to non-smokers [14]. The present study shows that smoking intensity influences SAF levels as well. SAF levels were highest in heavy smokers compared to light smokers, whereas no significant difference was found between heavy and moderate smokers.

Hoonhorst et. al. found a positive association between the amount of pack-years smoked and higher SAF levels among patients with chronic obstructive pulmonary disease indicating a dose-dependent effect [12]. Ohkuma et. al. examined the association between tobacco smoking and glycaemic control in type 2 diabetes patients and reported a significant increase in HbA1c levels with higher smoking intensity [26].

While Cerami et. al. have shown AGEs to be present in aqueous extracts of tobacco, Nicholl et. al. found higher AGEs levels in the lenses and blood vessels of tobacco smokers, demonstrating that tobacco smoke is an exogenous source of AGEs [15,27]. Tobacco smoke is associated with increased systemic oxidative stress, which may in turn contribute to exogenous AGEs formation as well [28]. AGEs have negative effects on insulin sensitivity, which might be due to increased oxidative stress and inflammation [29,30]. In addition, several AGEs can form cross-links within the vascular wall which results in impaired protein function and as a result increased vascular stiffness [31]. Finally, interaction of circulating AGEs with the AGEs receptor (RAGE) stimulates the production of pro-inflammatory cytokines, enhances oxidative stress [32] and causes endothelial dysfunction [33]. Therefore, tobacco smoke as an exogenous source of AGEs accumulation, may play a potential role in the underlying pathophysiological mechanism of type 2 diabetes [13] and a wide range of cardiovascular diseases, including (sub)clinical atherosclerosis, [34] coronary artery [35] and peripheral artery disease [36],

Using metabolomics data we have shown that cotinine N-oxide, a biomarker for environmental tobacco smoke exposure, is significantly associated with higher SAF levels in a group of individuals without diabetes. Therefore, urinary cotinine N-oxide might be used as an alternative way for questionnaires in demonstrating the effect of tobacco smoking on SAF. Remarkably, we found high levels of cotinine in never smokers, particularly in urine and saliva. Cotinine concentrations are four to five times higher in urine than in plasma and saliva which makes urine more eligible for detecting low-level exposure [37]. This could explain our detected sensitivity of 100% for cotinine N-oxide and supports our thoughts regarding the high levels of cotinine N-oxide in never smokers caused by secondhand smoking. Moreover, data from the third National Health and Nutrition Examination Survey (NHANES) suggest that around 90% of never smokers have detectable levels of serum cotinine [38]. It has been reported that the serum cut-off point to discriminate adult active smokers from non-smokers is 3 ng/ml [39]. Several explanations might be given for the fact that we observed high levels of cotinine in non-smokers. Firstly, in Qatar and the Middle-East, many public areas (such as restaurants) in the Qatar and the Middle-East still allow smoking, increasing the exposure to secondhand smoking in non-smokers. Secondly, we cannot exclude the possibility of heavy secondhand smoke exposure for some of the individuals, as well as the fact of misclassification of subjects smoking status according to their self-reports (e.g. smokers who occasionally smoke might be in fact considered as active smokers). In the study from Benowitz et. al, some of the individuals who reported to be a non-smoker had cotinine levels similar to levels of active smokers [39]. According to the cotinine levels reported in our study, it is likely that at least a proportion of non-smokers were in fact active smokers. Thirdly, racial/ethnic differences in the rate of metabolism of nicotine and cotinine have been described [40]. This supports our thoughts about secondhand exposure in (non-caucasian) non-smokers having a slower nicotine metabolism, and as a result, have elevated cotinine levels.

Fourthly, exposure to thirdhand smoke might also be an explanation for the high cotinine levels observed in non-smokers. Thirdhand smoke is generally considered to be residual tobacco smoke left on (indoor) surfaces such as sofas and wallpapers. Finally, several kinds of nutrition, such as vegetables and black tea, contain small amounts of nicotine but these levels are almost negligible.

In addition to active smoking, we were able to demonstrate that secondhand smoking is associated with higher SAF levels as well. Although we did not find all groups to be significantly different, a marked increase in SAF levels with higher exposure to secondhand smoking was observed. From these results, we learn that secondhand smoking can be added to the other determinants associated with SAF as demonstrated in our previous study [14].

Secondhand smoke is a combination of sidestream smoke and mainstream smoke which is exhaled smoke by an active smoker [41]. Sidestream smoke contains a relatively higher concentration of unfiltered toxic gases and small respirable particles than mainstream smoke [42] and is thus potentially more hazardous compared to the smoke which is directly inhaled by active smokers [43]. Accumulating evidence has shown that secondhand smoking increases the risk of type 2 diabetes [44] and coronary artery disease [45,46] by increasing oxidative stress, systemic inflammation and endothelial dysfunction [28,47]. Furthermore, tobacco smoking may contribute to insulin resistance [48] and has been demonstrated to be associated with a higher risk of pancreatitis and pancreatic cancer suggesting that chemical compounds in tobacco smoke may have direct toxic effects on pancreatic β cells [49]. In a similar way as active smoking, it is highly likely that enhanced AGEs formation is involved in the pathophysiological pathway by which secondhand smoking increases the risk of both type 2 diabetes and cardiovascular diseases.

To our knowledge, this is the first study showing that quitting smoking has a reversible effect on SAF levels. We observed that SAF Z-scores of former smokers gradually decreased with increasing years since quitting smoking, even after adjusting for BMI.

After around 15 years of smoking abstinence, SAF Z-scores had returned to levels of never smokers. Ohkuma et. al. have shown a decrease in HbA1c levels in subjects with type 2 diabetes as the years since quitting smoking increased [26]. Since HbA1c is actually an intermediate product of glycation, our findings might be considered in line with their results.

Our data provide some indication that at least part of the effect of tobacco smoking on AGEs accumulation is reversible in individuals who quit smoking, indicating a beneficial effect of smoking cessation on both metabolic and glycaemic control.

It has been reported that smoking cessation reduces the risk of type 2 diabetes [50] and cardiovascular disease [51]. Paradoxically, smoking cessation is commonly associated with subsequent weight gain [52] which in turn might attenuate the beneficial effects of smoking cessation. Several potential mechanisms may contribute to a lower risk of type 2 diabetes and cardiovascular disease associated with smoking cessation, including improved insulin sensitivity and lipoprotein levels as well as reduced inflammation [53–55]. Considering the role of increased AGE accumulation as a consequence of tobacco smoking in its association with type 2 diabetes and cardiovascular disease, it is likely that reduced formation and accumulation of AGEs may result in reduced type 2 diabetic and cardiovascular risk. However, our hypothesis needs to be confirmed by future translational studies.

Our study has some strengths and limitations. Firstly, part of this study is based on a large number of participants, resulting in a good statistical power and the ability to perform analysis for different smoking status and classes. A limitation of the LifeLines Study is the potential misclassification of individuals with regards to their smoking status as we cannot completely rule out misreporting of smoking habits or history. Secondly, we were not able to stratify the analyses for diabetes status since the number of subjects with type 2 diabetes was too small.

A limitation of the QMDiab study is the small number of study participants which may have caused a reduced statistical power in finding significant associations. Since the QMDiab study did not include questions regarding secondhand smoking, we were not able to assess any correlation with cotinine biomarkers.

## Conclusions

This study clearly demonstrated that secondhand smoking is associated with higher SAF levels whereas smoking cessation led to a gradual normalization of SAF levels as the years since smoking abstinence increase. Moreover, we have demonstrated that urinary cotinine N-oxide, a biomarker for environmental tobacco smoke, is significantly associated with higher SAF levels and might therefore be used as an alternative way for questionnaires to examine the effect secondhand smoking on SAF. These findings should be taken into consideration in future studies on SAF or using SAF as a screening tool for populations at risk for diabetes and cardiovascular diseases.

## Supporting information

S1 Table Sensitivity and specificity for cotinine markers in current smokers (QMDiab Study). Missing saliva collection (n = 44), plasma collection (n = 16), urine collection (n = 14). Missing smoking data (n = 10).
(DOCX)
