# Supplementary material for: The association between various smoking behaviors, cotinine biomarkers and skin autofluorescence, a marker for advanced glycation end product accumulation
Source: PLoS One. 2017 Jun 20;12(6):e0179330. doi: 10.1371/journal.pone.0179330 (PMC5478117; doi:10.1371/journal.pone.0179330)
Supplement: S1 Table — Missing saliva collection (n = 44), plasma collection (n = 16), urine collection (n = 14). Missing smoking data (n = 10). (DOCX) [file pone.0179330.s001.docx]

| **Cotinine marker** |  |  |
| --- | --- | --- |
| Cotinine saliva (n=330) | N (% total) / N (% current smoker) / N (% former or non-smokers) | 62 (19.4%) / 27 (93.1%) / 35 (12.1%) |
|  | sensitivity/specificity | 93.1% / 88.0% |
|  | AUC (CI) | 0.91 (0.85, 0.96) |
| Cotinine plasma (n=358) | N (% total) / N (% current smoker) / N (% former or non-smokers) | 61 (17.6%) / 30 (96.8%) / 31 (9.8%) |
|  | sensitivity/specificity | 96.8% / 90.2% |
|  | AUC (CI) | 0.94 (0.89, 0.98) |
| Cotinine urine (n=360) | N (% total) / N (% current smoker) / N (% former or non-smokers) | 89 (25.5%) / 32 (97.0%) / 57 (18.0%) |
|  | sensitivity/specificity | 97.0% / 82.1% |
|  | AUC (CI) | 0.90 (0.85, 0.94) |
| Cotinine N-oxide urine (n=360) | N (% total) / N (% current smoker) / N (% former or non-smokers) | 148 (42.4%) / 33 (100.0%) / 115 (36.4%) |
|  | sensitivity/specificity | 100.0% / 63.8% |
|  | AUC (CI) | 0.82 (0.77, 0.87) |
| Hydroxy-Cotinine urine (n=360) | N (% total) / N (% current smoker) / N (% former or non-smokers) | 29 (8.3%) / 14 (42.4%) / 15 (4.7%) |
|  | sensitivity/specificity | 42.4% / 95.3% |
|  | AUC (CI) | 0.69 (0.58, 0.80) |

**S1 Table.** **Sensitivity and specificity for cotinine markers in current smokers (QMDiab Study)**

Missing saliva collection (n=44), plasma collection (n=16), urine collection (n=14). Missing smoking data (n=10).
